# Pathophysiology and surgical decision-making in central cord syndrome and degenerative cervical myelopathy: correcting the somatotopic fallacy

**DOI:** 10.3389/fneur.2023.1276399

**Published:** 2023-11-17

**Authors:** Husain Shakil, Carlo Santaguida, Jefferson R. Wilson, H. Francis Farhadi, Allan D. Levi, Jared T. Wilcox

**Affiliations:** ^1^Division of Neurosurgery, St. Michael's Hospital, University of Toronto, Toronto, ON, Canada; ^2^McGill University Health Center, Montreal Neurological Institute-Hospital, McGill University, Montreal, QC, Canada; ^3^Department of Neurosurgery, University of Kentucky, Lexington, KY, United States; ^4^Department of Neurological Surgery, The Miami Project to Cure Paralysis, University of Miami Miller School of Medicine, Miami, FL, United States

**Keywords:** cervical spine, spinal cord injury, central cord syndrome, myelopathy, neurosurgery, operative management, surgical intervention, clinical outcome

## Abstract

Our understanding of Central Cord Syndrome (CCS), a form of incomplete spinal cord injury characterized by disproportionate upper extremity weakness, is evolving. Recent advances challenge the traditional somatotopic model of corticospinal tract organization within the spinal cord, suggesting that CCS is likely a diffuse injury rather than focal lesion. Diagnostic criteria for CCS lack consensus, and varied definitions impact patient identification and treatment. Evidence has mounted for early surgery for CCS, although significant variability persists in surgical timing preferences among practitioners. A demographic shift toward an aging population has increased the overlap between CCS and Degenerative Cervical Myelopathy (DCM). Understanding this intersection is crucial for comprehensive patient care. Assessment tools, including quantitative measures and objective evaluations, aid in distinguishing CCS from DCM. The treatment landscape for CCS in the context of pre-existing DCM is complex, requiring careful consideration of pre-existing neurologic injury, patient factors, and injury factors. This review synthesizes emerging evidence, outlines current guidelines in diagnosis and management, and emphasizes the need for ongoing research to refine our understanding and treatment strategies for this evolving patient population.

## Introduction

Central cord syndrome (CCS) is a form of incomplete spinal cord injury (SCI) characterized by disproportionate upper extremity weakness compared to the lower extremities. Over the last decade, our understanding of CCS has undergone a paradigm shift (1, 2). Recent preclinical studies challenge the longstanding belief of a somatotopic representation of the corticospinal tract within the spinal cord—the presumed basis for our mechanistic understanding of CCS (3). Similarly, recent clinical trials have demonstrated the need to re-evaluate practices relating to timing of surgery (4). Moreover, the epidemiology of patients presenting with acute CCS is changing, largely due to an aging population which has led to a greater incidence of CCS in patients with pre-existing degenerative cervical myelopathy (DCM) (5, 6).

This primary objective of this review is to describe recent advances in our understanding of CCS, with a focus on pathophysiology and surgical decision making, while comparing this condition to DCM.

## Central cord syndrome pathophysiology: a historical perspective

**Figure 1:**
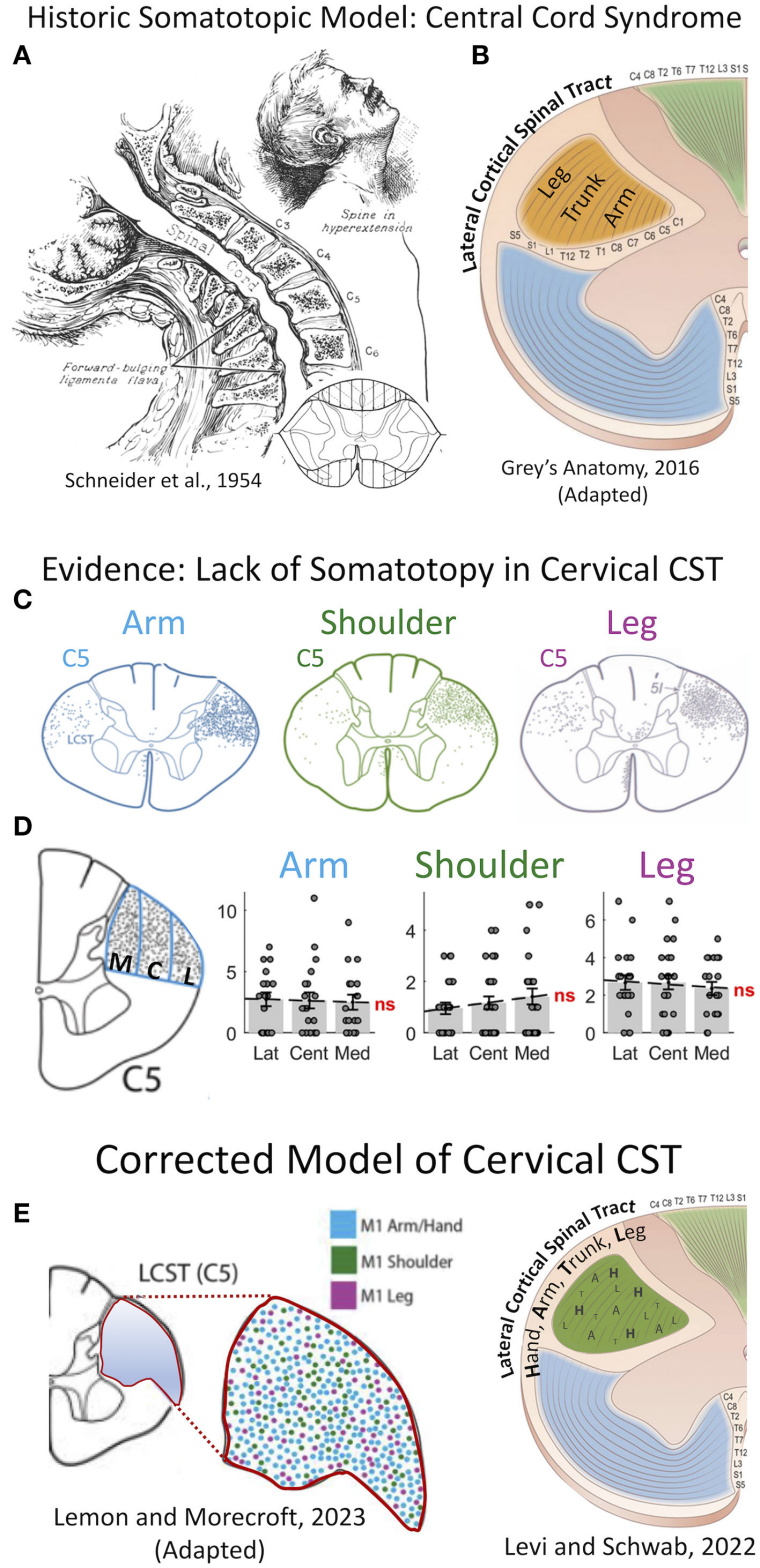
Re-appraisal of pathophysiology of central cord syndrome (CCS) with error of historic models of somatotopic organization in the spinal cord. **(A)** Central cord syndrome as classically described in 1954 by Schneider et al. as traumatic hyper-extension in a stenotic cervical spinal column leading to an incomplete spinal cord injury affecting the upper more than the lower extremities (6). **(B)** The prevailing theory for the pathophysiology of CCS, until recently, was that the cortical spinal tract (CST) has a lamellar organization in the cervical spinal cord with the upper extremity axons coursing in the most mesial portion, thereby leading to greater injury of the descending control of the arm and hand, and preservation of the leg. This theory was propagated in most—if not all—major texts of anatomy and neurology until recently, including this image from Gray's Anatomy (41^st^ Ed, 2016). **(C–E)** This long-held hypothesis of CST somatotopy has been shown inaccurate by careful work in non-human primates. **(C)** The CST fibers serving the upper extremity and lower extremity course throughout the cross-sectional area of the tract. **(D)** Quantification of axon density shows no difference in mesial to lateral sections. **(E)** A recent model from Lemon and Morecraft (7) demonstrates a complete lack of somatotopic organization with random dispersion of CST axons in the cervical enlargement in primates. C5, 5^th^ cervical level; CCS, central cord syndrome; CST, corticospinal tract; M1, primary motor cortex; ns, not significant. (A) This figure is protected by Copyright, is owned by The JNS Publishing Group, and is used with permission only within this document. Permission to use it otherwise must be secured from The JNS Publishing Group. Full text of the article containing the original figure is available at thejns.org. **(C–E)** © 2022 The authors, CC BY-NC-ND 4.0 (http://creativecommons.org/licenses/by-nc-nd/4.0/); **(F)** © 2022 Roberto Suazo.

Central cord syndrome is a form of incomplete SCI that affects upper extremity more than lower extremity strength. Initially described by Schneider et al. in the 1950s (6), the pathology results from anteroposterior compression of the spinal cord from “forward bulging ligamentum flavum” in combination with “osteophyte formation…on cervical vertebral bodies in senile spines” typically after hyperextension injuries (Figure 1). Remarkably, it was known for a century prior to this description that traumatic cervical injury superimposed on spinal stenosis may occur without significant bony fractures yet result in central hematomyelia (8).

In primates, the corticospinal tract (CST) is responsible for fine motor control of the extremities (9, 10). An injury disproportionately affecting the upper extremity was historically presumed to be due to damage in a specific region of the CST. This led to the so-called “central cord syndrome,” with the presumption that the mesial portion—or concentric lamellar layers—specifically contain axons innervating the upper extremity tracts. This has long been the historic model of spinal cord structural organization, comprising mesial-to-lateral concentric organization of the corticospinal tract, respectively arranged for rostral-to-caudal motor control (Figure 1).

## Evidence challenging spinal cord motor somatotopy

Several studies have provided strong evidence against a somatotopic organization of the CST within the spinal cord of humans and non-human primates using advanced imaging, tractography, and tracing studies (Figure 1) (9, 11–13). The specific data has been expertly reviewed by Levi and Schwab (3). The classic model of descending motor control of the limbs as a single synapse from cortex to contralateral alpha motor neuron is likely an oversimplification. Studies have shown that axons of the CST form collaterals and bifurcations in the cervical spinal cord of non-human primates, with nearly twice as many fibers projecting ipsi-, contra-, and bi-laterally as found in the originating dorsolateral funiculi (11, 14). This suggests that a major contributor to CCS is due to these decussations and interneuron connections in the cervical enlargement. Humans also have two-and-a-half more axonal fibers in the CST as compared to old-world monkeys, and greater manual hand dexterity due to greater direct cortex to alpha motor neuron connections (10, 15). A more modern view of spinal cord injury syndromes such as CCS or Bell's cruciate paralysis, is that of a diffuse injury causing disproportionate injury to axons of the hand and upper-extremities due to the disproportionate density of these axons within the CST of the cervical cord. This is distinctly different than the prior theory of a focal targeted lesion within the CST. The growing evidence of complexity and networked pathways in the cervical spinal cord are only now being mapped in detail with fluorescent tracing and trans-neuronal synaptic mapping (10). Ongoing research is required to improve understanding of this pathophysiological circuitry to better understand the pathophysiology of CCS and potentially devise novel treatment strategies.

## Controversies in diagnostic criteria for central cord syndrome

Today, there is no widely accepted or validated diagnostic criteria used to define CCS. The case definition of CCS was initially provided by Schneider et al. in 1954 (6) as disproportionate motor impairment of the upper limbs, neurogenic bladder dysfunction, and sensory loss as a result of injury to a region of the cervical spinal cord. Today, patients presenting with acute cervical injury are assessed using the International Standards for Neurologic Classification of Spinal Cord Injury worksheet (16). Upper extremity motor scores (UEMS) are graded on a 5-point scale per limb for elbow flexors, wrist extensors, elbow extensors, finger flexors and finger abductors. This is summed to provide a 50-point total UEMS. The hip flexors, knee extensors, ankle dorsiflexors, long toe extensors, and ankle plantar flexors are similarly graded to provide a 50-point total lower extremity motor score (LEMS). A systematic review and survey conducted by the European Multicenter Study about Spinal Cord Injury (EM-SCI) reviewed diagnostic criteria for CCS and found 7 different disease descriptions. The authors conducted a meta-analysis of patients presenting with CCS and found a mean difference in UEMS and LEMS of 10.5. The authors proposed a quantitative definition of CCS as a minimum 10-point difference in UEMS relative to LEMS (17, 18). Other authors have used a minimum 5-point difference to define CCS (4). Guideline and validation studies are needed to arrive at a consensus diagnostic criterion for CCS. This will allow for more consistent identification and treatment of patients suffering from this disease.

## Variability in surgical management of central cord syndrome

Over the last decade, the role of early surgery for CCS has been revisited. Historically, management of CCS was largely driven by outcomes reported in Schneider's original papers on CCS published in the 1950s. In his reports, Schneider described poor surgical outcomes based on intradural dissection with or without myelotomy, and concluded that “decompressive laminectomy is futile” (6). Lasting impacts have remained regarding timing of surgery and the notion that CCS is a distinct entity from SCI. The nature and techniques of surgical decompression, and perioperative care have significantly advanced since the original studies. Surgical decompression is now widely accepted as appropriate in the treatment of CCS, with early decompression known to be safe and cost-effective (19). Despite this, an international survey and consensus review demonstrated tremendous variability in surgeon preferences regarding timing of surgery for CCS (20). When presented with a case of a 65-year-old patient with traumatic CCS and underlying cervical stenosis, a similar proportion of surgeons would opt for ultra-early surgery (2–4 h, 13.5%) as for delayed surgery (6 weeks, 16%). Subsequent studies found that 55% of patients presenting with acute traumatic CCS were managed non-operatively (21), and more than 55% of surgeons who operate on traumatic CCS do so after 24 h from presentation (22).

## Early decompression for central cord syndrome

Recent work from Badhiwala et al. (4) suggests a role for early surgery for CCS to improve outcomes. A propensity-matched meta-analysis on 186 patients with CCS (5-point threshold) drawn from 3 prospective multicenter studies [North American Clinical Trials Network (NACTN) SCI Registry (23); Surgical Timing in Acute Spinal Cord Injury Study (STASCIS) (24); and National Acute Spinal Cord Injury Study (NASCIS) III) (25)] demonstrated significantly greater upper limb recovery (+2.3 UEMS; 95% CI 0–4.5) following early (< 24 h) vs. late (≥24 h) surgery. Moreover, subgroup analysis revealed this difference was largely due to ASIA C patients who had total motor recovery of +9.5 points (UEMS + LEMS, 95% CI 0.5 – 18.4) while ASIA D patients showed no difference. These results suggest that early surgery likely promotes recovery in certain subpopulations and explains why motor improvements may not have been observed in earlier studies (19). Separate meta-analyses, and cohort studies have also shown benefit for early surgical decompression in cases of CCS and cervical SCI (26–28). These research findings suggest that early surgery should strongly be considered and discussed with patients presenting with acute CCS.

## A growing overlap between central cord syndrome and degenerative cervical myelopathy

The demographics of patients suffering from SCI is changing with the aging population. Multiple studies have noted an increasing incidence of SCI among the elderly in the last two decades, with fall injuries more than doubling (29, 30). Wilson et al. noted these higher rates to be due to increases in incomplete cervical SCI, with CCS representing the most common form (29). As such we can expect an increasing number of patients with CCS over time. From 2009 to 2012, ~11,975 cases of acute CCS were found to have presented to emergency departments across the United States (21).

The rise in North American life expectancy will likely be associated with an increased incidence of age-related disease. DCM represents age-related degeneration of the spine and will likely be a driving force in the incidence of acute CCS. Gait dysfunction and imbalance associated with DCM is known to increase the propensity for falls, which is currently the most common mechanism of injury for CCS (21). In the coming years, we can expect more overlap between the population of patients with DCM and CCS. As such, a complementary understanding of the two diseases is necessary to provide comprehensive care to patients suffering from CCS with pre-existing DCM.

## Assessment of central cord syndrome in the context of degenerative cervical myelopathy

Assessment and management of mild forms of CCS remain a challenge. The gold standard for grading myelopathy today is the modified Japanese orthopedic association scale (mJOA). However, assessment with the mJOA have been found to have poor inter-rater reliability for scoring of upper extremity function (31). This presents a unique challenge to patients presenting with CCS with pre-existing DCM. In the context of a low-impact trauma, such as a fall from standing height, it can be difficult to discern the contributions of mild spinal cord injury superimposed on progressive chronic myelopathy. Similarly, following patients with mild DCM can be challenging as they often report falls, and it is unclear if subtle changes relate to the natural history of progressive DCM, or an intervening injury resulting in mild CCS. Utilization of objective assessment tools may offer some insight into recovery trajectory and differentiation of CCS from DCM.

Numerous radiologic and quantitative outcome measures have been described for monitoring symptoms and response to treatment for DCM (32–34). Certain intrinsic signal changes identified on sagittal or axial-based MRI been shown as predictive of motor recovery in SCI (35, 36), CCS (4, 19, 37), and DCM (33, 34). The size of signal change has been found to be predictive of greater functional improvement than having received early decompressive surgery (27, 28, 36); however, underpowering represents a common issue that limits subgroup analysis. Quantitative clinical measures used for assessing lower extremity motor function include the 10 or 30-meter walk tests (38), and GAITrite analyses (32). Assessment tools for upper extremity motor deficits include the modified GRASSP (39), grip dynamometry (40), and the QuickDASH (41). In the setting of mild DCM, these tools are likely to provide a more nuanced assessment of patient symptoms beyond the widely used modified Japanese Orthopedic Association scale (42, 43). Many surgeons now advocate following DCM patients with simple grip dynamometry, and/or patient reported outcomes in addition to mJOA for clinical decision-making (44). More work is needed to reliably delineate CCS from DCM and validate the use of different radiologic and quantitative clinical assessment tools to improve management of acute CCS in the setting of pre-existing myelopathy.

## Treatment of central cord syndrome in the context of degenerative cervical myelopathy

DCM represents a state of aberrant physiology due to both static and dynamic factors that contribute to chronic spinal cord compression and injury (1, 45). Static factors that reduce the cross-sectional area of the spinal canal include congenital stenosis, age-related intervertebral disc degeneration, spondylosis, and ossification of the posterior longitudinal ligament (OPLL) (46–48). Chronic compression due to static factors, stenosis, and deformation of blood vessels is thought to lead to local hypoperfusion. This has been proposed to lead to a cascade of hypoxic ischemia, and blood spinal cord barrier disruption, with subsequent microglia and macrophage mediated neuroinflammation (49, 50). Dynamic factors that have been implicated in the pathogenesis of DCM include regional instability with resultant degenerative spondylolisthesis due to anteroposterior subluxation and horizontal translation of the vertebral bodies, as well as spondylosis causing a pathological range of motion at diseased segments (46, 48, 51). Current guidelines recommend early surgery for moderate-severe DCM (mJOA ≤ 14), and in cases of neurologic deterioration in patients with mild DCM (mJOA > 14). Surgery is thought to address static factors through decompression of the cervical cord, and dynamic factors through arthrodesis of the pathological segments.

Treatment of acute CCS in preexisting DCM represents a significant challenge. Further research is needed to delineate the additional impact of an acute injury to a spinal cord that has been chronically hypoperfused, as in the case of DCM. It is currently unclear exactly how pre-existing DCM impacts the recovery trajectory for patients with acute CCS. Emerging therapies may help address this state of combined acute and chronic injury. Durotomy is now re-emerging as a potential treatment option for SCI and been suggested to provide greater reduction in intramedullary pressure compared to decompression alone (52). Neuroprotective agents such as riluzole, and stem cell based regenerative therapies are being investigated as possible additional treatment strategies for acute CCS and DCM (53). Additional adjuvant treatments include mean arterial pressure (MAP) augmentation, and spinal cord perfusion pressure (SCPP) augmentation. There is evidence to suggest that maintaining MAP ≥ 85 mmHg correlates with improved neurologic recover in SCI, which is in keeping with current guidelines (PMID 25669633). Alternatively, monitoring SCPP using a lumbar thecal catheter, and maintaining levels ≥ 50 mmHg has been shown to independently be associated with improved neurologic recover in patients with SCI (54).

## Illustrative cases

We present two surgical cases of acute CCS with pre-existing DCM that illustrate the complexity of decision making for patients with acute CCS and DCM.

**Figure 2:**
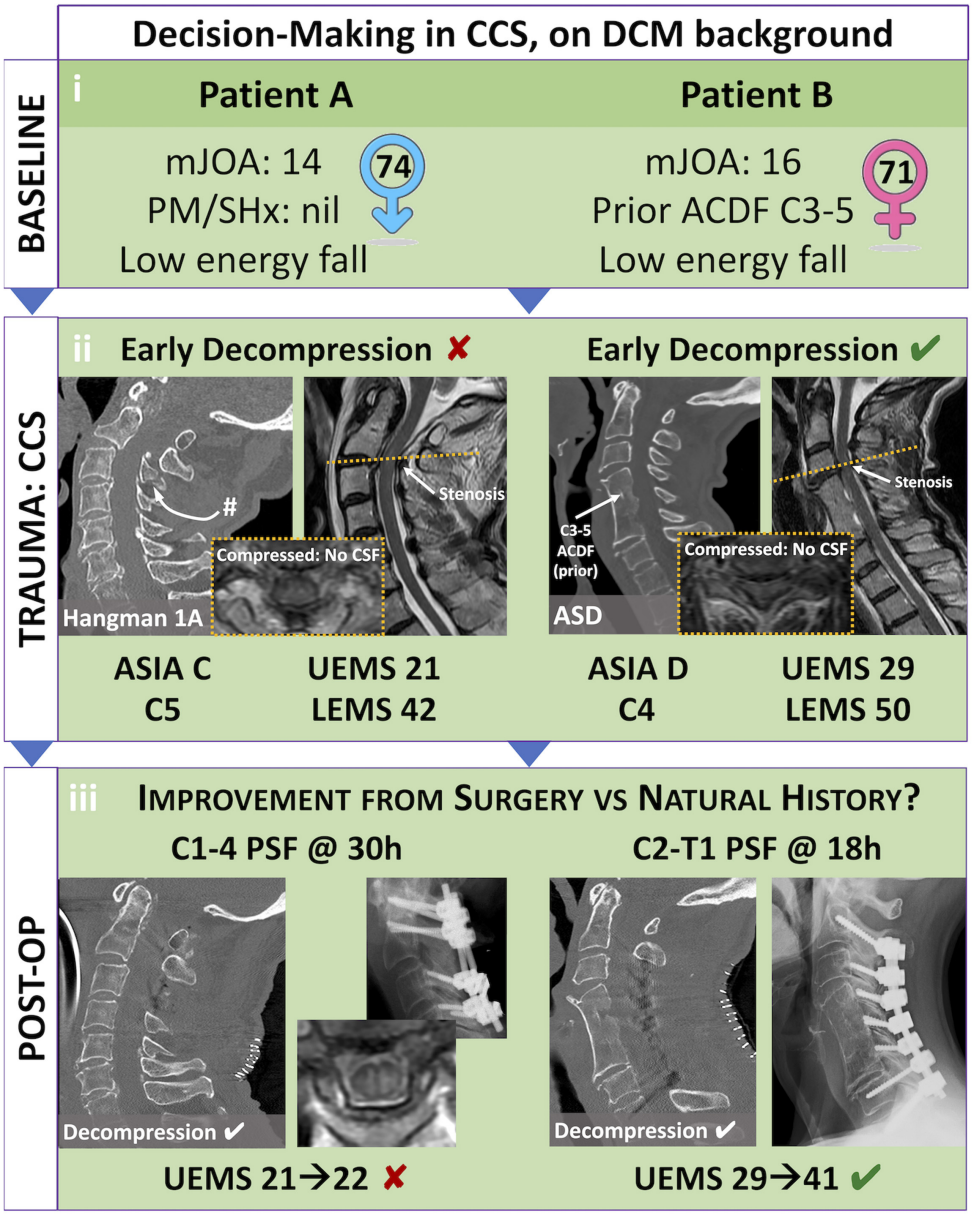
Illustrative case study of Central Cord Syndrome (CCS) on pre-existing Degenerative Cervical Myelopathy (DCM): evaluating best patient selection in elective and emergent settings. Patient selection for elective decompression of DCM and emergent decompression of CCS can prove difficult. **(i)** While both previously healthy, Patient A is a healthy 74-year-old-male who has not received decompression for his moderate DCM (mJOA 14), while Patient B is a healthy 71 year-old-female who was offered a two-level ACDF for mild DCM (mJOA 16). **(ii)** Following falls, both these patients presented with CCS that included significant weakness and sensory changes. Patient A had a C5 level ASIA C grade injury, with inability to move his arms and hands against gravity. Patient B had a C4 level ASIA D grade injury, with ability to move her arms and hand against gravity but without meaningful strength. **(iii)** Each patient had a successful decompression of their cervical compression; however, Patient A was delayed in receiving surgery due to inadequate identification of spinal cord injury and consultation to a specialized Spine Surgeon. Patient B had emergent surgery in less than 24 h. Based on current evidence, we suggest that—while both patients are candidates—Patient A may have a greater predictive benefit from elective decompression of chronic moderate DCM, as well as emergent (< 24 h) surgical decompression of acute traumatic AIS C grade CCS. #, fracture; ACDF, anterior cervical decompression and fusion; ASD, adjacent segment disease; ASIA, American Spinal Cord Injury Association Impairment Scale; C4/5, 4/5^th^ cervical level; CCS, central cord syndrome; DCM, degenerative cervical myelopathy; mJOA, modified Japanese Orthopedic Association scale; PM/SHx, past medical and surgical history; PSF, posterior spinal decompression and fusion; UEMS/LEMS, Upper/Lower Extremity Motor Score.

Patient A, a 72-year-old male presented to our level 1 trauma centre after falling five stairs. At baseline, he had mild clumsiness of his hands and difficulty with stairs, with an mJOA 14/18 (42). The patient had no notable past medical history and presented with dense weakness of 2/5 in bilateral upper extremities and 4+ to 5/5 power in bilateral lower extremities. He was given a working diagnosis of C5 ASIA C CCS with UEMS of 21 and LEMS of 42. Consultation to the spine service was delayed as managing physician(s) understood CCS to be non-surgical. After consultation to the spine team, mean arterial pressure goals were initiated, and magnetic resonance imaging of the cervical spine obtained. Imaging revealed significant compression at C1-2 with cord signal change due to a type 1A hangman's fracture (Figure 2ii). Surgical decompression was ultimately completed 30 h post-injury, with some delay due to systemic COVID-19 related resource restrictions. A C1-4 posterior instrumented fusion and decompression was completed without complication (Figure 2iii). Unfortunately, the patient's UEMS and ASIA grade did not meaningfully improve post-operatively.

Patient B, a 71-year-old-female presented after pre-syncopal fall while intoxicated. She was known for prior C3-5 anterior discectomy and fusion for DCM with mJOA 16/18 and had been asymptomatic since this remote operation. The patient was given a working diagnosis of C4 ASIA D incomplete SCI with UEMS 29 and LEMS 50. Rapid consultation to the spine service was initiated, and subsequently mean arterial pressure goals were initiated, along with acquisition of spinal imaging. Computed tomography revealed no significant fracture, but magnetic resonance imaging of the cervical spine demonstrated compression cephalad to the prior fusion at C3/4. The patient underwent urgent surgical decompression within 24 h of injury. Post-operatively she improved significantly with UEMS 41 (+12) at 6-week follow-up.

Recognition of the surgical indications and need for urgent intervention are paramount for patients' presenting with CCS and DCM. As illustrated by the above cases (Figure 2), key factors that influence patient trajectory include: (i) the nature of their pre-existing DCM (ii) the nature of their acute CCS and (iii) the timing of surgery. Patient A is slightly older, with worse baseline myelopathy that was untreated. Evidence suggests that older age and longer duration of symptoms predict poor outcomes after surgery for DCM, and this may extend to acute CCS with pre-existing DCM (55–57). Patient A also presented with a worse AIS grade, and signs of micro-instability on imaging suggesting a higher severity of trauma (58). Patient A underwent surgical decompression after 30 h post-injury. An earlier decompression may have provided improved SCPP to minimize the impact of secondary ischemic injury. All factors when taken together likely contributed to the minimal recovery after surgery. In contrast Patient B sustained a similar mechanism of injury but had a previously treated milder form of DCM. They also presented with a higher AIS-grade CCS and underwent decompression within 24 h of injury. These factors when taken together likely contributed to the patient's significant neurologic recovery.

## Summary and closing

We have summarized the recent advances in our understanding of the pathophysiology and management of CCS and provided context to the growing overlap of this disease with DCM. Evidence against a somatotopic arrangement of the CST in the cervical cord suggest CCS is represents a diffuse injury to the cervical cord, likely with similarities to DCM. Assessment of CCS in the background of DCM is challenging, however the increased adoption of quantitative assessment tools for baseline upper and lower extremity function of patients with DCM, may aid in delineating acute injury from chronic. Furthermore, a consensus definition of CCS is required among clinicians to appropriately identify patients and provide equity in care. In keeping with guidelines, we recommend early surgery for patients presenting with CCS. Table 1 provides a summary of assessment and treatment recommendations discussed in this review. Ongoing research is required to better understand the pathophysiology of CCS, how this may be altered in the setting of chronic injury and apply this knowledge to design novel treatment strategies for the changing patient population. Furthermore, quantifying the relationship between pre-existing DCM, and severity of CCS with recovery trajectory will help provide guidance to patients and clinicians faced with this devastating condition.

**Table 1:** Summary of current assessment tools and guidelines for treatment of acute central cord syndrome (CCS) and degenerative cervical myelopathy (DCM).

	**CCS**	**DCM**
Assessment	ISNCSCI worksheet **UEMS—LEMS** **≥** **5**	Myelopathy grade mJOA Upper extremity function Modified GRASSP, grip dynamometry, QuickDASH Lower extremity function 10/30 m walk test, GAITrite analysis
Timing of surgery	Surgery ≤ 24 h	mJOA **≤** **14** Surgery at earliest available date mJOA **>** **14** Surgery at earliest date OR initial conservative management w/ surgery if any deterioration

mJOA, modified Japanese Orthopedic Association scale; ISNCSCI, International standards for neurologic classification of spinal cord injury; UEMS/LEMS, Upper/Lower Extremity Motor Score.

## Author contributions

HS: Conceptualization, Writing – original draft, Writing – review & editing. CS: Data curation, Writing – review & editing. JRW: Writing – review & editing. HFF: Writing – review & editing, Resources. ADL: Supervision, Writing – review & editing. JTW: Conceptualization, Data curation, Supervision, Validation, Visualization, Writing – original draft, Writing – review & editing.
